# Trade in medicines and the public's health: a time series analysis of import disruptions during the 2015 India-Nepal border blockade

**DOI:** 10.1186/s12992-017-0282-0

**Published:** 2017-08-22

**Authors:** Abhishek Sharma, Shiva Raj Mishra, Warren A. Kaplan

**Affiliations:** 1 Precision Health Economics, Boston, MA USA; 2 Center for Global Health and Development, Department of Global Health, Boston University School of Public Health, Boston, MA USA; 3 Nepal Development Society (NEDS), Bharatpur-10, Chitwan Nepal

**Keywords:** Trade and health, Border blockade, Health diplomacy, Access to medicines, Nepal, India

## Abstract

**Background:**

Nepal was struck by devastating earthquakes in April–May 2015, followed by the India-Nepal border blockade later that year.

**Methods:**

We used the United Nations Commodity Trade Statistics (UN Comtrade) database to analyse exports of various health commodities from India to Nepal from January 2011–September 2016. We used time-series regressions of trading volume vs. unit price to ask how well Nepal’s trading history with India prior to the earthquake and blockade was able to predict unit prices of health commodities imported into Nepal during and after the earthquake and the blockade. Regression residuals were used to quantify the extent to which the blockade impacted the price of healthcare commodities crossing into Nepal.

**Results:**

During the blockade period (September 2015-early February 2016), the volume of all retail medicines traded across the India-Nepal border was reduced by 46.5% compared to same months in 2014–2015. For medical dressings, large volumes were exported from India to Nepal during and shortly after the earthquakes (May–June 2015), but decreased soon thereafter. During the earthquake, the difference between observed and predicted values of unit price (residuals) for all commodities show no statistical outliers. However, during the border blockade, Nepal paid USD 22.3 million more for retail medicines than one would have predicted based on its prior trading history with India, enough to provide healthcare to nearly half of Kathmandu’s citizens for 1 year.

**Conclusion:**

The India-Nepal blockade was a geopolitical natural experiment demonstrating how a land-locked country is vulnerable to the vagaries of its primary trading partner. Although short-lived, the blockade had an immediate impact on traded medicine volumes and prices, and provided a large opportunity cost with implications for public health.

**Electronic supplementary material:**

The online version of this article (doi:10.1186/s12992-017-0282-0) contains supplementary material, which is available to authorized users.

## Background

Understanding the relationship between international trade and health is critical to managing access to medicines in the global public health “ecosystem” [1]. Dependence on international trade is likely to increase, especially in those low and middle income countries with limited local production of health technologies and this is particularly so for health commodities related to non-communicable diseases [2, 3].

There have been long-standing debates focused on aligning trade policies with human rights and improving access to essential health technologies, but these have been limited primarily to the global intellectual property rights (IPR) regime and those discontented with it [4–7]. Despite efforts to identify appropriate indicators and mechanisms to continuously monitor for any potential public health risks associated with trade policies, there is a general lack of empirical evidence on how international trade, particularly the cross-border movement of health technologies, enhances or deteriorates population health [8].

In recent years, countries in the European Union (EU) have been repeatedly accused of blocking legitimate trade of Indian-manufactured generic medicines by detaining these consignments while in-transit to low-income countries [9]. In Cuba, the trade embargo imposed by the United States has been reported to risk many lives [10]. Similarly the Israel’s blockade of Gaza strip has been often reported to have caused chronic shortage of medicines at healthcare facilities in Gaza [11]. The global economic crisis led to increase in mortality rates due to poor quality of care in Greece [12].

Nepal receives nearly 60% of its overall imports of all commodities – including most medicines and health commodities – from India [13]. Under the provision of Articles 6 and 7 in the 1950 Indo-Nepal Treaty of Peace and Friendship and its subsequent versions, there is an open border between the two countries allowing free movement of people and trading goods [14]. In the spring of 2015, Nepal was struck by devastating earthquakes that left the country – with already a weak public health system - in a dire need for rehabilitation health care [15]. What then soon followed after the earthquakes was an unfortunate blockade of the India-Nepal border for several months [16]. There were media reports on disrupted supply of petroleum, gas and essential medicines, affecting civilians’ lives and also forcing hospitals to shut all services expect the emergency services [16]. Public health programs such as vaccinations were affected in districts bordering with India. However, this was not the first time the border was completely shut. In the 1990s, a border blockade lasted for one and half years.

We have previously described the background to the 2015–2016 blockade faced by Nepal which resulted from a domestic conflict and a diplomatic standoff with India [17]. However, its impact on health has never been documented. Prior research has focused on the negative impact of multi-year economic sanctions on morbidity and mortality in various countries with little or no attention paid to comparisons with pre-sanction conditions (see ‘Research in Context’ below). In this paper, we explore the impact of this blockade on the Nepalese population’s access to many health care commodities which Nepal imported from India, by analyzing real-world trade data on unit prices and volumes before, during and after the blockade. This is the first empirical study on the impact of the India-Nepal blockade on medicines access using quantitative time-series trade data.

**Table:** 

RESEARCH IN CONTEXT
*Evidence before this study*
In the 1990s, critics began to argue that economic sanctions indiscriminately and unjustly targets poor and innocent elements of society. We searched PubMed, Web of Science and GoogleScholar without date restrictions for English language sources on February 4th and 6th, 2017, using several search combinations with the terms ‘Trade’, ‘border’, ‘blockade’, ‘embargo’, ‘medicines’, ‘access to medicines’, and ‘access to health commodities’ to find articles on primary assessment of impact of border blockade on access to healthcare commodities. Previous research has explored the negative impact of long-lasting (i.e., multi-year) economic sanctions on the public’s health in Cuba, Haiti, Iraq, Yugoslavia, Iran, Syria and the occupied Palestinian territory. However this evidence is primarily focused on the impact of such sanctions on human behavior and psychology as well as morbidity and mortality, and relies on case studies, media reports and other qualitative forms of information. Furthermore, several articles encompass the debates focused on aligning trade policies with human rights and improving access to essential health technologies, but have been limited primarily to the global intellectual property rights regime. When the literature focuses on specific medicines, it emphasizes medicine shortages and increased retail prices within the impacted country, with no comparisons to the pre-sanction period.
*Added value of this study*
Using comprehensive import-export data (2011–2016), we studied the relatively short-lived trade sanction of several months (September 2015 to early-February 2016) and focused on the time course of medicine trade before and after the sanctions. Although Nepal relies upon India as its largest trading partner, we found that the unit price of a “basket” of all retail medicines increased as the blockade took effect and trade volume decreased. For most of the blockade, the increase in unit price was far in excess of what the pre-sanction trade relationship between price and volume would have predicted. While one could also have predicted that such a short-term (5 month) trade blockade imposed by India in late 2015 would also have little impact on medicine prices and, by implication, medicine access, the amount of extra money Nepal paid for this diminished supply of medicines, even over this relatively short time period, indeed had significant opportunity costs. Nonetheless, for some medicines, e.g., insulin, various antibiotics, there was little obvious impact of the trade blockade on unit price. One might have predicted that Nepali earthquakes of April–May 2015 would impact quantities and prices of medicines exported into Nepal from its largest traded partner India, but that was not the case.
*Implications of all the available evidence*
Besides media and health advocacy organizations often document the health-related experiences of local populations during trade disruptions, there is little empirical evidence on the impact of trade disruptions on access to health commodities. Trade policies cannot be separated from population health, and governments and health professionals must facilitate appropriate and effective policy coherence between the two. This study demonstrates what it means for one country to be, in effect, dependent on a single exporter. In principle, nations will engender public health risk if they are not regularly importing biomedical commodities from other nations worldwide and are most vulnerable if they have no or limited local production.

## Methods

The Nepal earthquakes were in April and May 2015 and the India blockade lasted from September 2015 to early-February 2016 [18]. Nepal depends on medicines imports from India to meet nearly 60–70% of its healthcare needs and Nepal’s domestic pharmaceutical manufacturers entirely depend on imports for raw materials [19]. Using time-series regression of trading volume vs. unit price data, and analyses of the resulting unit price residuals, we studied how well Nepal’s prior trading history with India could predict prices paid by Nepal during and after the blockade.

### Data sources and definitions

#### Data source

National law usually requires that importers and exporters of goods report particulars of their trade transactions to customs offices for the purposes of collection of duties and taxes and for health, environmental, and/or other control and statistical purposes. The national statistical agencies then submit this trade information to the United Nations (UN) Statistical Division, which then performs quality testing and compiles these records into the UN Commodity Trade Statistics (UN Comtrade) database [20]. The UN Comtrade database records commodity imports and exports by the trading dyad (importing and exporting country), by value (U.S. dollars) and volume (net weight in kilograms).

Imports into Nepal are recorded as CIF (cost insurance and freight), e.g., transaction value of the goods plus the value of services performed to deliver goods to the Indian-Nepali border. Exports from India are FOB (free on board), e.g., transaction value of the goods and the value of services performed to deliver goods to the border of the exporting country (India). This may represent a 10 to 20% difference in value [21]. We empirically confirmed this by regressing the yearly monetary values since 2009 of all retail medicines (Comtrade commodity code 54) exported to Nepal from all its reporting trading partners against the value of the identical commodity as reported being imported by Nepal from these identical country trading partners. If the dyadic symmetry is perfect, we would expect a slope of 1.0 for the regression. We found that there is a 13% difference in value (slope of 1.13: r^2^ = 0.93; data in Additional file 1), consistent with Comtrade assertions. We left this difference uncorrected in our monthly data analysis. Furthermore, less than 1% of monthly data had missing values, typically for net weight.

### Classification of commodities

We used the Harmonized Commodity Description and Coding System (HS), an international nomenclature for the classification of products, to define the medical technology commodities [22].

For purposes of this study, we used 4-digit or 6-digit HS classifications, as appropriate for the questions asked, to analyze cross-border trade of the following commodities: “Medicaments consisting of mixed or unmixed products for therapeutic or prophylactic use, put up in measured doses or in forms or packings for retail sale” (i.e., retail medicines in dosage form); “Medicaments; containing penicillins, streptomycins or their derivatives, for therapeutic or prophylactic uses, packaged for retail sale” (i.e., retail penicillins and streptomycins); “Medicaments; containing insulin (but not containing antibiotics), for therapeutic or prophylactic uses, packaged for retail sale” (i.e. retail insulin); “Medicaments; containing antibiotics (other than penicillins, streptomycins or their derivatives), for therapeutic or prophylactic uses, packaged for retail sale” (i.e., retail antibiotics aside from penicillin and streptomycin); “Dressings, adhesive; and other articles having an adhesive layer, packed for retail sale for medical, surgical, dental or veterinary purposes” (i.e., retail medical dressings). See Table 1: columns 1 and 2.

**Table 1:** Net price residuals (in 2015 USD) for health commodities during India-Nepal border blockade

Commodity (1)	Comtrade commodity number (2)	No. Standardized Residuals >2 during Sept 2015-March 2016 (3)	Net Residual (Constant 2015 U.S. Dollars) Sept 2015-March 2016 (4)
Medicaments, therapeutic, prophylactic use, in dosage form	3004	3	+ 22.31E + 06
Penicillins and streptomycins derivatives, in dosage form	300410	0	+ 2.15E + 06
Antibiotics “not elsewhere specified”, in dosage form	300420	0	+ 0.803E + 06
Insulin, in dosage form	300431	0	- 121,690
Medical dressings	300510	0	- 47,452

### Statistical analyses

We obtained monthly UN Comtrade data for exports of the various medical commodities from India to Nepal from January 1, 2011 to September 1, 2016 [20]. Then, we extracted data from January 1, 2011 to just before the earthquake (April 1, 2015) and calculated, from the value (USD) and volume (kg) data, the month-specific unit price (USD/g) of a given health commodity (listed in Table 1) exported from India to Nepal. We corrected the monthly unit price for constant 2015 U.S. dollars. We empirically determined the best-fit of this relationship between unit price and volume over the January 1, 2011 to April 1, 2015 time period using CurveExpert 1.40® curve fitting software [23]. The software is packaged with a series of linear and non-linear regression models and ranks the model fits.

Using the empirical relationship between volume and unit price between January 1, 2011 and April 1, 2015, we then predicted what the unit price of a given commodity should be for each month over the entire 5 year time course, including both earthquakes and the blockade subsequent to April 1, 2015. We next calculated the monthly unit price residuals for a given commodity, i.e., difference between the observed and predicted unit prices for any month and then standardized these residuals by dividing each residual by the standard deviation of all residuals. A good guideline for standardized residuals is a standardized residual larger than about ±2 should be investigated as a potential outlier, since that would only be expected to occur randomly about 1% of the time. Multiple standardized residuals >2 suggest something quite unusual.

We defined the trade blockade as running from September 1, 2015 to March 1, 2016. Over this time period, we multiplied the monthly residual unit price for a health commodity by the total weight of that health commodity exported from India into Nepal in that month. With a positive residual, this gives an estimate of how the money paid by Nepal for that particular commodity is greater than what one would have expected from Nepal’s prior trade history with India. We summed up all these values for the defined blockade period.

## Results

### Trade time series

#### Retail dosage form medicines

Between 2011 and late-2016, over 95% of all monthly exports of retail medicines in dosage form (Comtrade code 3004) into Nepal from all reporting countries originated as exports from India. Countries such as the Netherlands (0.8% of exports), the United Kingdom (0.5%) and the EU-27 countries make up the difference.

The empirical relationship between unit price and volume (kg) is always non-linear for all the medical technology commodities. As the export weight (kg) decreases, not surprisingly, unit prices increase. The relationship is typically defined as a power or exponential function. See Additional file 1: Figure S1.

**Fig. 1:**
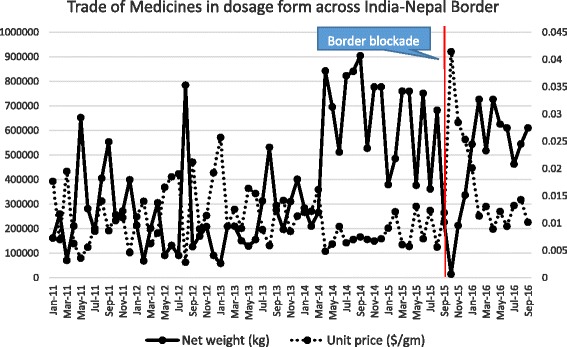
Net weight (kilograms: *solid line*) and unit price (USD/g: *dotted line*) of all retail medicines imported from India into Nepal before and after the border blockade (*vertical line*)

Figure 1 plots the time series (January 1, 2011-Sept 1, 2016) of exports from India to Nepal by volume (kilograms) and unit price (USD/g) for all retail “medicines for prophylactic and therapeutic purposes in dosage form” (Comtrade Code 3004). There is a general and fluctuating trend in monthly volume (mean 210,592 kg) from January 2011–February 2014 emphasized by a ‘step’ increase in March 2014 to a new mean monthly volume of 696,012 kg during April 2014–August 2015. After August 2015, there is a sharp decrease and a rebound in late 2015-early 2016. The inverse relationship between unit price and volume is also illustrated in this Figure. There is no clear signal in volume or unit price during the earthquake (April–May 2015). Yet, during the blockade period, the volume of all retail medicines traded across the India-Nepal border was 46.5% lower than that during same months in 2014–2015. See Additional file 1: Table S2(a).

#### Retail dosage form insulin

**Fig. 2:**
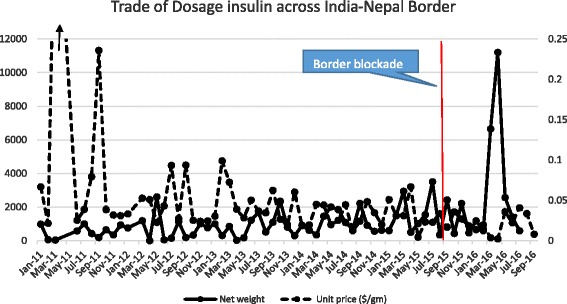
Net weight (kilograms: *solid line*) and unit price (USD/g: *dotted line*) of insulin products imported from India into Nepal before and after the border blockade (*vertical line*)

Figure 2 plots the time series (January 1, 2011-September 1, 2016) of exports from India to Nepal (in kilograms) and unit price (USD/g) for all retail insulin in “dosage form” (Comtrade Code 300431). There are large price fluctuations in 2011 but generally less so over time emphasized by a sharp increase in trade in April 2016, when the blockade ended. During both the earthquake and the blockade period itself, it is difficult to see any large fluctuations in trade in retail dosage insulin. See Table 1.

#### Medical dressings

**Fig. 3:**
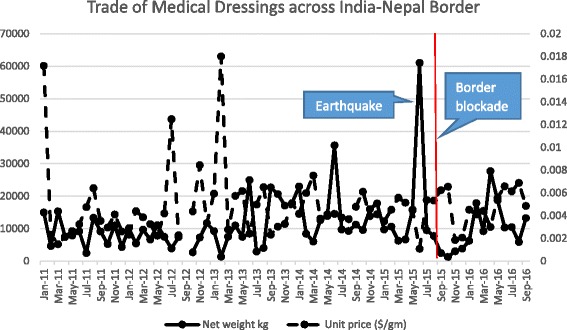
Net weight (kilograms: *solid line*) and unit price (USD/g: *dotted line*) of medical dressings imported from India into Nepal before and after the border blockade (*vertical line*)

Figure 3 plots the time series (January 1, 2011 - July 1, 2016) of exports from India to Nepal (in kilograms) and unit price (USD/g) for all “medical dressings, with adhesive backing” (Comtrade Code 300510). There is a large volume export signal (May–June 2015) and an almost immediate drop and a rebound in late 2015-early 2016. During the border blockade, the exports of medical dressings dropped by 84.9% and 53.5%, compared to the quantities that were exported in May–June 2015 and the average exports during the same months in 2014, respectively. See Additional file 1: Table S2(b).

### Analysis of residuals

#### Retail dosage form medicines

The standardized residuals for all retail medicines “for prophylactic and therapeutic purposes in dosage form” (Comtrade Code 3004) show no statistical outliers (greater than −2 and +2) during the earthquake period April–May 2015. See Additional file 1: Figure S2. However, the residuals during the blockade (November 2015–January 2016) all have positive standardized scores between 2.6 and 3.7 as the observed unit price for these particular months is statistically greater than the predicted unit price. Although the individual residual values are small (i.e., less than USD 0.015 per gram), the volumes traded are not. Between September 2015 and February 2016, we estimate that Nepal paid USD 22.3 million more for retail dosage form medicines than one would have predicted based on its prior trading history (January 2011–April 2015) from India (Table 1, column (5)). This "overpayment" of USD 22.3 million during the blockade is 7.6% of the total value of all dosage medicines (USD 293 million) exported from all countries into Nepal during pre-earthquake period between January 2011 and March 2015.

#### Medical dressings

The standardized residuals for all “medical dressings” (Comtrade Code 300510) reveal that exports from India into Nepal of medical dressings lacked statistical outliers either during the earthquake or the blockade. The volume of trade in medical dressings increases around the time of the 2015 earthquake and decreases during the blockade, coming back to pre-blockade levels in the first half of 2016. As expected, when the volume of trade decreases, the unit price increases. We note two short-lived unit price fluctuations in 2011 and 2012. A single monthly shipment during the blockade (October 2015) showed a very strong negative residual (observed unit price less than predicted), exceeding the outlier boundary. See Additional file 1: Figure S3.

Table 1 shows the results of identical analyses for various other medicine and non-medicine health commodities. As an example of the calculations, see Additional file 2 for the penicillin and streptomycin exports into Nepal. In brief, the price of exports from India to Nepal of penicillins and streptomycins in dosage form as well as various unspecified antibiotics in dosage form were about what one would have been expected during the blockade, i.e., net positive residuals (Table 1, column (4)) as none of the residual values were statistical outliers during this same time period. Insulin and medical dressings showed a relatively trivial amount less than one would have predicted from the prior years of trade with no unusual standardized residuals.

## Discussion

India is the main supplier of all medicines to Nepal. Between 2011 - mid 2016, generally over 95% of Nepal’s imports of all retail dosage medicines (both prophylactic and therapeutic) were from India and this is the commodity with the largest number of unit price outliers (November 2015–January 2016). Specifically, during the 2015–2016 time periods prior to, and after, the earthquakes and blockade, over 90% of the weight of retail dosage form medicines originated in India with correspondingly much smaller amounts from the EU-27 and Switzerland (Additional file 1: Figure S4). The sole exception is the blockade month of October 2015 when total exports of retail dosage form medicines into Nepal fell to very low levels and the EU-27 contributed about 40% of this total weight. This overall pattern is similar to the export of retail antibiotics of various types (Additional file 1: Figures S5-S6). We note the relative lack of export data for the EU-27 with regard to penicillin and streptomycin (Additional file 1: Figure S5), although when monthly comparisons are available, India is clearly the predominant supplier, as it is for medical dressings (Additional file 1: Figure S7).

While media and health advocacy organizations have often documented the health-related experiences of patients and local populations during trade disruptions, there still appears to be little quantitative evidence on the impact of trade disruptions on access to medicines at such times.

Using time series and residual analysis, we found that there was no significant reduction in the volume of medical commodities exported from India to Nepal during the earthquake period (See Figs. 1, 2 and 3), but during the blockade, the impact on volume and price was substantial. The unit price of retail dosage medicine increased as the blockade intensified but the three consecutive (i.e., monthly) residuals that are statistical outliers during the blockade (Additional file 1: Figure S2) are unusual.

We note that the use of time series regressions that analyze residuals for ‘outliers’ is a method commonly used with various degrees of sophistication in disciplines as diverse as econometrics [24–26], public health [27, 28] and climatology [29].

The earthquake primarily affected northern regions of Nepal [30, 31], so the movement of medicines and other health commodities across the southern India-Nepal border was not disrupted. Rather, during the earthquake, India supplied medicines and other essential commodities to Nepal through its India-Nepal border as a humanitarian crisis response [32]. However, the only spike in Indian exports to Nepal during the earthquake was the influx of medical dressings in May 2015 which we infer is a ‘marker’ for this humanitarian response. See Fig. 3.

Notwithstanding, the relatively brief border blockade several months later had a serious impact on volume and price of retail dosage medicines at the India-Nepali border and this clearly had consequences in-country [33, 34]. Using our modeling technique, we found that the impact of the blockade was immediate on imports of retail dosage medicines into Nepal from September 2015–March 2016, which were about 46% less than same period in 2014–2015 (Additional file 1: Table S2). The lower trade volumes for retail dosage medicine imports into Nepal led to considerably increased trade prices for medicines for several months, far in excess of what would have been predicted prior to the blockade (See Table 1). One might be surprised at this from a political and human rights viewpoint, given that Nepal relies on India as its primary trading partner. Compared to what one would have predicted the unit import price to be for “retail medicines in dosage form”, Nepal paid in excess of USD 22.3 million more for the medicines it imported during the 5 months September 2015 – early-February 2016, USD 15.9 million of which was paid for between November 2015–January 2016.

To put this in context, these 22.3 million U.S. dollars has an opportunity cost that could have financed basic yearly healthcare for 557,500 people based on Nepal’s 2014 annual per capita healthcare spending of USD 40.00 [35], and this is about half the population of Nepal’s largest metropolitan area, Kathmandu. Put another way, Nepal could have purchased enough doses of pentavalent (DPT-HepB-Hib) vaccine, at GAVI-negotiated prices [36], to provide a complete vaccination course to 3.2 million children i.e. four times Nepal’s annual birth cohort.

Importers may have been unable to negotiate lower prices given smaller purchase volumes during the blockade. Health commodities at times were flown into Nepal from Indian or via South-east Asian cities [37]. Moreover, reports suggest that over 400 trucks containing medicinal products and raw material awaited border clearance for over 1–2 months; this not only have implications for medicine availability but also risked the degradation, deterioration, or transformation of these inappropriately stored medicines [37].

However some medicines, namely various antibiotics and retail insulin in dosage form, showed little major change in trade volume across the blockade boundary (See Table 1). This result for insulin is, in principle, encouraging. In fact, during the study period, the majority of retail insulin by volume exported into Nepal originated from India (Additional file 1: Figure S8) but this data cannot tell us if the insulin was manufactured by Indian companies or made under license in India for multinationals [38]. We note, however, that diabetes and NCDs in general do have a lower funding profile and health system response in Nepal compared to infectious diseases so perhaps the unchanging, but low volume of insulin trade during the blockade is not surprising. In 2010, only 0.7% of Nepal’s total budget was for non-communicable (NCD) prevention and control, and its overall per capita health spending (5.6% of gross domestic product) is one of the lowest in the world [3]. Diabetes receives a tiny fraction of this budget despite its growing burden and associated complications [3].

There are several limitations to our analysis. The overall retail ‘*Medicaments, therapeutic, prophylactic use, in dosage form*’ commodity basket in the Comtrade dataset probably contains hundreds of different medicines. Therefore, we are unable to say which medicines were contributing to the excessively high unit prices for the blockade months. Data on “retail insulin” is not dis-aggregated by type (i.e., pen, vial, analog, human). Also, Comtrade data is voluntary and it is merely “recommended” that medicines that are donated as part of humanitarian aid are included.

Any increases in India-derived medicine prices represents a lower limit of the increase in patient prices in Nepal as some of the Nepali in-country supply-chain costs (taxes, margins, mark-ups, and delivery costs) would be higher than usual during the blockade in part due to acute shortage of fuel [16, 34, 39]. We do not have data on availability and prices of any of these Comtrade biomedical commodities in the public- or private-sector health facilities inside Nepal during the blockade so trade data are only surrogate indicators of in-country access.

Previous research has explored the negative impact of long-lasting (i.e., multi-year) economic sanctions on the public’s health in Cuba, Haiti, Iraq, Yugoslavia, Iran, Syria and the occupied Palestinian territory [40–46]. Academics do not need long-term evaluations of economic sanctions to understand its health impacts. For instance, in Iraq, during just 4 months, the sanctions showed a greater influence on the hazard of dying than did any of the traditional risk factors. Among Iraqi children, the sanctions period accounted for a four-fold increase in the risk of a child dying [47].

Health cannot be separated from trade, and governments need appropriate and effective policy coherence between the two. Impacts on health from a political disaster such as the border blockade can be reduced through sustainable diplomacy [48]. Although the blockade was relatively short-lived, health ministers should be communicating with Prime Ministers and/or foreign affairs and home ministries to manage health effects of trade blockade. Therefore, in general, it is imperative for the health sector to engage in broader health-related trade aspects beyond the current focus on the IPR regime [4–7], and be able to facilitate corrective policy measures. However there appears no special provisions to protect trade of health-related commodities during such diplomatic border blockades. One suggestion would be to establish some sort of “express cross-border fast track” that provide immunity to health-related trade from any such diplomatic blockade.

Healthcare professionals must not think that trade is too complicated and perhaps does not affect them. In fact, health professionals are well positioned to sense subtle fluctuations in supply of health technologies and therefore should familiarize themselves with health-related trade in their local context. This would empower them to become more vocal about their role in ameliorating the health effects of any trade disruptions. The general public, even less aware of the connection between the trade and health systems, has a role to play as well. During such disasters, the public’s general reaction is to overstock and hoard medicines, creating shortages. Therefore, public awareness and not overstocking medicines can help remove pressure on an already-constrained supply chain. In this regard, a surveillance system on trade volume and flow of medicine (e.g., those on the WHO Essential Medicines Lists) should be built and made publicly available for monitoring and research.

This manuscript is based on a ‘natural experiment’ which is likely to happen more frequently than supposed, so it is certainly possible that, given the appropriate dataset, one could look at other ‘economic shocks’, as it were - although they are likely to also be retrospective in nature. It may also be possible to look prospectively, most usefully for land-locked countries with no or limited local production of relevant commodities and only 1–2 key trading partners. More generally, this work sheds some light on what it means for one country to be, in effect, dependent on a single exporter. In principle, and irrespective of a trans-border blockade, nations will engender public health risk if they are not regularly importing biomedical commodities from other nations worldwide and are most vulnerable if they have no or limited local production [2]. When the number of suppliers declines, the ability of individual suppliers to raise prices can be increased. Several policy solutions could be investigated. For one, Nepal’s government procurement policy might be improved to obtain medicines from its suppliers at the lowest possible price or, more generally, to achieve the best value for money. Vigorous competition among suppliers can help governments attain this objective.

## Conclusion

The India-Nepal blockade was a geopolitical natural experiment imposing conditions on a land-locked country that is obviously vulnerable to the vagaries of its primary trading partner. Although short-lived, the blockade had an immediate impact on traded medicine prices and on Nepali finances. We were limited in the quantity and type of data available during the blockade yet the effect on the day-to-day health of Nepali citizens cannot be denied [34, 49].

After the 2015–2016 blockade, it seems that diplomatic relations between Nepal and India have not returned its pre-blockade levels even after 2 years [50]. Political considerations will always impact health and humanitarian concerns. Our retrospective analysis of medicine cross-border trade during the blockade found that the money spent by Nepal on medicines over and above that predicted from 4 years of prior trade had significant opportunity costs with respect to the public’s health. The more subtle role of trade in the absence of a trade perturbation will impact health systems in ways that are complex but that should be monitored.

## Additional files

Additional file 1: Time-series analyses of trade in health commodities, 2011-2016. (PDF 801 kb)

Additional file 2: Residual calculation for penicillin and streptomycin exports. (XLSX 54 kb)
