# Supplementary material for: Trade in medicines and the public's health: a time series analysis of import disruptions during the 2015 India-Nepal border blockade
Source: Global Health. 2017 Aug 22;13:61. doi: 10.1186/s12992-017-0282-0 (PMC5568715; doi:10.1186/s12992-017-0282-0)
Supplement: Supplementary file 1 — Time-series analyses of trade in health commodities, 2011-2016. (PDF 801 kb) [file 12992_2017_282_MOESM1_ESM.pdf]

Additional file - 1

Definition: Net weight in UN Comtrade data

Particularly for medicines in liquid form (i.e., insulin), the definition of net weight adopted for goods contained in a package for retail sale, is supposed to exclude the "…outer package, but include the inner package for retail packing…" but for liquids the net weight of goods is the weight of the liquid, "… which should exclude the packaging, even if for retail packing." [1]

Difference in the way COMTRADE records imports and exports

In the UN Comtrade data, imports are recorded as CIF (cost insurance and freight) which include the transaction value of the goods, the value of services performed to deliver goods to the border of the exporting country, and the value of the services performed to deliver the goods from the border of the exporting country to the border of the importing country. The exports are recorded as FOB (free on board) which include the transaction value of the goods and the value of services performed to deliver goods to the border of the exporting country. This, according to the UN International Merchandise Trade Statistics, usually represents a 10-20% difference in the trade values reported by the Reporters reporting imports and those reporting exports. To confirm this empirically, we regressed monetary values of all retail medicines (COMTRADE commodity code 54) exported to Nepal from all its trading partners (as "Reporters) since 2009 against the value of the same commodity imported by Nepal (as "Reporter") from these identical country "Partners". If the dyadic symmetry is perfect, we would expect a slope of 1.0 for the regression. We found that there is a 13 percent difference in value (slope of 1.13: $r^2 = 0.93$), consistent with COMTRADE assertions (Figure S1).[1]

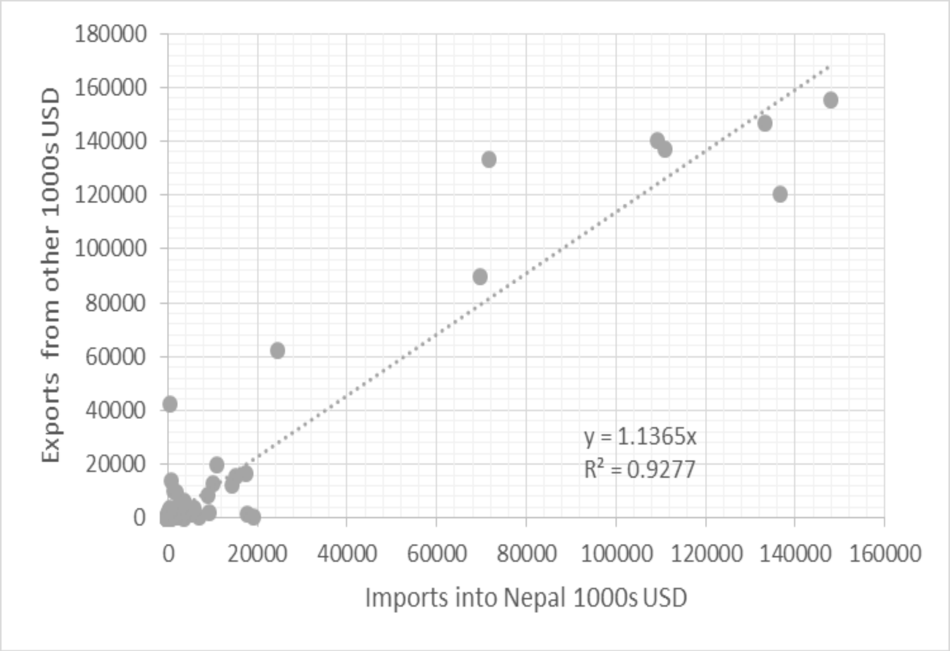

Figure S1: Regression of monetary value of medicines exports to Nepal as reported by other countries versus the value of medicines imports in Nepal as reported by Nepal.

Table S1: Impact of Nepal earthquakes on volumes of health-related trade imported in Nepal

**Table S1(a): Impact of Nepal earthquakes on 'all medicines' trade volume**			
**Period description**	**Exporting Country**	**Importing Country**	**Volume traded (KGs)**
Earthquake period			
April 2015	India	Nepal	760,138
May 2015	India	Nepal	376,533
**Mean monthly volume traded during Nepal earthquakes (i.e. April 2015 – May 2015)**			**568,336**
One year prior to Nepal earthquakes			
April 2014	India	Nepal	842,960
May 2014	India	Nepal	696,012
**Mean monthly volume traded during same months an year ago**			**769,486**
**Reduction in cross-border trade volumes during Nepal earthquakes period compared to same months an year ago (%)**			**201,150 (26.14%)**

**Table S1(b): Impact of Nepal earthquakes on 'medical dressings' trade volume**			
**Period description**	**Exporting Country**	**Importing Country**	**Volume traded (KGs)**
Earthquake period			
April 2015	India	Nepal	6,649
May 2015	India	Nepal	15,853
**Mean monthly volume traded during Nepal earthquakes (i.e. April 2015 – May 2015)**			**11,251**
One year prior to Nepal earthquakes			
April 2014	India	Nepal	12,632
May 2014	India	Nepal	14,409
**Mean monthly volume traded during same months an year ago**			**13,521**
**Reduction in cross-border trade volumes during Nepal earthquakes period compared to same months an year ago (%)**			**2,270 (16.8%)**

Table S2: Impact of Indo-Nepal border blockade on volumes of health-related trade imported in Nepal

**Table S2(a): Impact of Indo-Nepal border blockade on 'all medicines' trade volume**			
**Period description**	**Exporting Country**	**Importing Country**	**Volume traded (KGs)**
Blockade period			
September 2015	India	Nepal	227,080
October 2015	India	Nepal	14,971
November 2015	India	Nepal	213,183
December 2015	India	Nepal	337,080
January 2016	India	Nepal	544,023
February 2016	India	Nepal	726,883
**Mean monthly volume traded during blockade period (i.e. September 2015 – February 2016)**			**343,870**
One year prior to Blockade Period			
September 2014	India	Nepal	9045,66
October 2014	India	Nepal	527,483
November 2014	India	Nepal	777,348
December 2014	India	Nepal	777,336
January 2015	India	Nepal	379,931
February 2015	India	Nepal	486,315
**Mean monthly volume traded during same months an year ago**			**642,163**
**Reduction in cross-border trade volumes during blockade period compared to same months an year ago (%)**			**298,293 (46.45%)**

**Table S2(b): Impact of Indo-Nepal border blockade on 'medical dressings' trade volume**			
**Period description**	**Exporting Country**	**Importing Country**	**Volume traded (KGs)**
Earthquake period			
May 2015	India	Nepal	15,853
June 2015	India	Nepal	61,028
**Mean monthly volume traded during earthquake period**			**5,813**
Blockade period			
September 2015	India	Nepal	227,080
October 2015	India	Nepal	14,971
November 2015	India	Nepal	213,183
December 2015	India	Nepal	337,080
January 2016	India	Nepal	544,023
February 2016	India	Nepal	726,883
**Mean monthly volume traded during blockade period**			**38,440**
One year prior to Blockade Period			
September 2014	India	Nepal	9045,66
October 2014	India	Nepal	527,483
November 2014	India	Nepal	777,348
December 2014	India	Nepal	777,336
January 2015	India	Nepal	379,931
February 2015	India	Nepal	486,315
**Mean monthly volume traded an year earlier during months same as the blockade period**			**12,491**
**Reduction in cross-border trade during blockade compared to the earthquake period (%)**			**38440.9 (84.9%)**
**Reduction in cross-border trade during blockade compared to the same months an year ago (%)**			**12491 (53.5%)**

Standardized residuals from predicting medicine import prices from trade

Figure S2 plots the standardized residuals for all "medicines for prophylactic and therapeutic purposes in dosage form" (Comtrade Code 3004). The boundaries of what standardized residuals would be considered statistical outliers (greater than -2 and +2) are shown. We see no statistical outliers during April-May 2015 (earthquakes). The residuals for November 2015-January 2016 all have positive standardized scores between 2.6 and 3.7 as the actual unit price for these particular months is statistically greater than the predicted unit price. Although the individual residual values are small (i.e., less than USD 0.015 per gram), the volumes traded are not. Between September 1, 2015 and March 1, 2016, we estimate that Nepal paid USD 22.3 million dollars more for dosage form medicines than one would have predicted based on its prior trading history (January 2011-April 2015) from India (see Table 1, column (5) in manuscript).

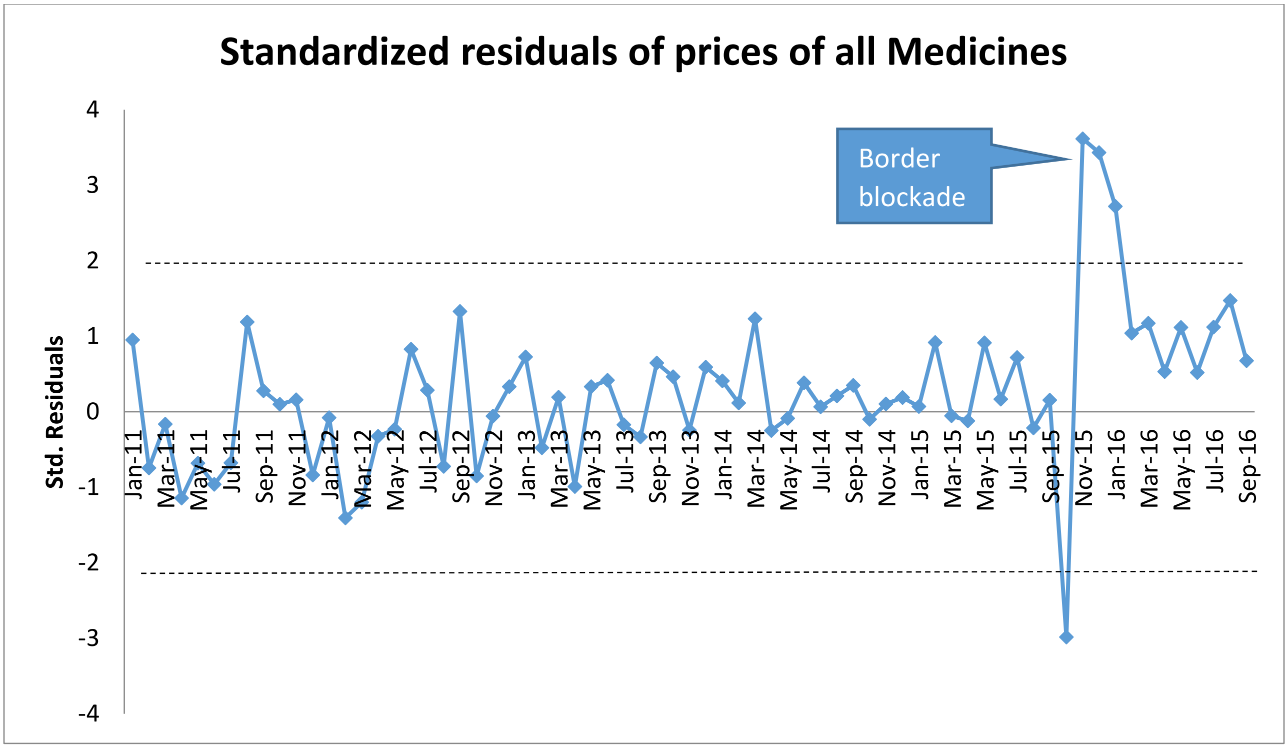

Figure S2. Standardized residuals of prices of all medicines imported from India into Nepal during the border blockade

Figure S3 plots standardized residuals for all "medical dressings" (Comtrade Code 300510). The outlier boundaries are shown. Exports from India into Nepal of medical dressings in July 2012 had a higher unit price than predicted but we see no such outliers either during the earthquake or the blockade. One monthly shipment during the blockade (October 2015) showed a very strong negative residual (unit price less than predicted) exceeding the outlier boundary.

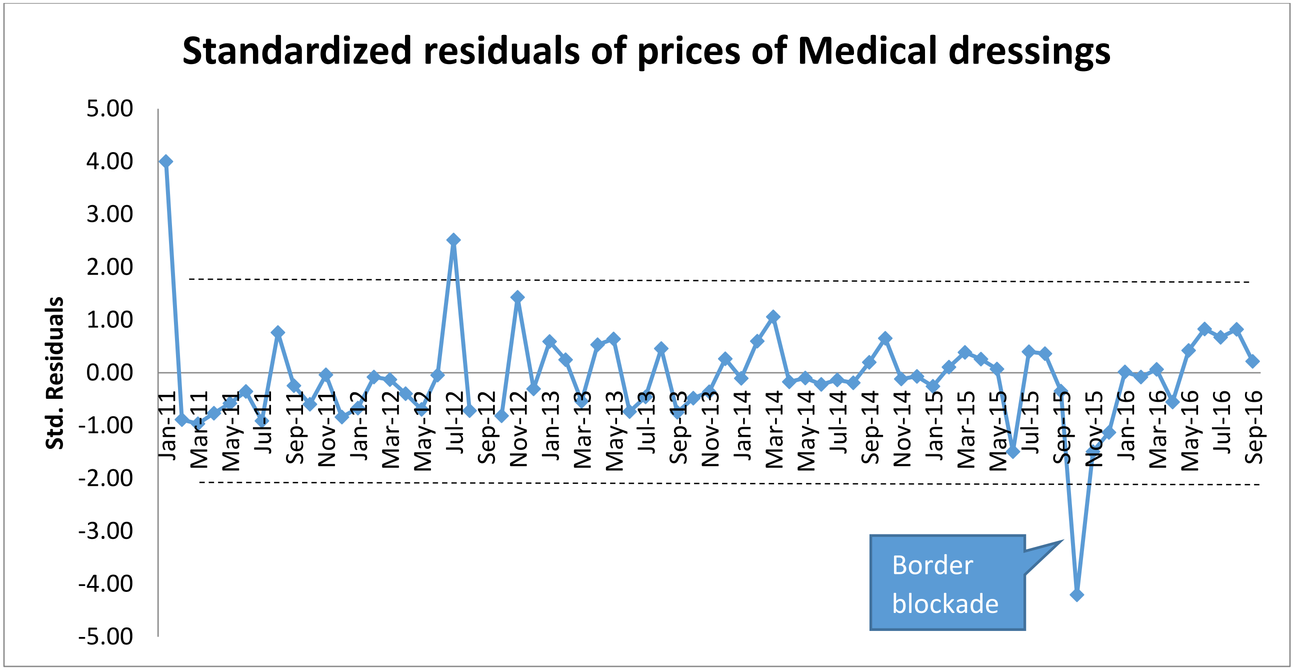

Figure S3. Standardized residuals of prices of medical dressings imported from India into Nepal during the border blockade.

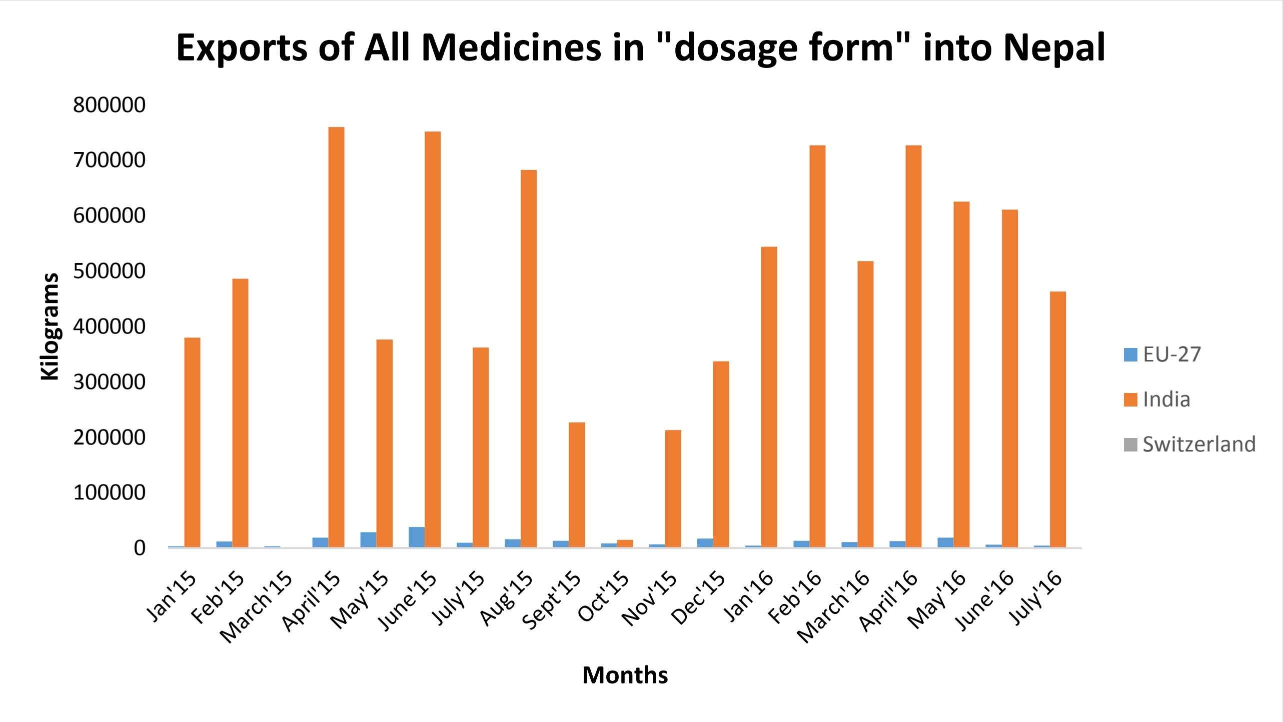

Figure S4. Monthly exports (by weight) of all medicines in dosage form into Nepal before, during and after border blockade

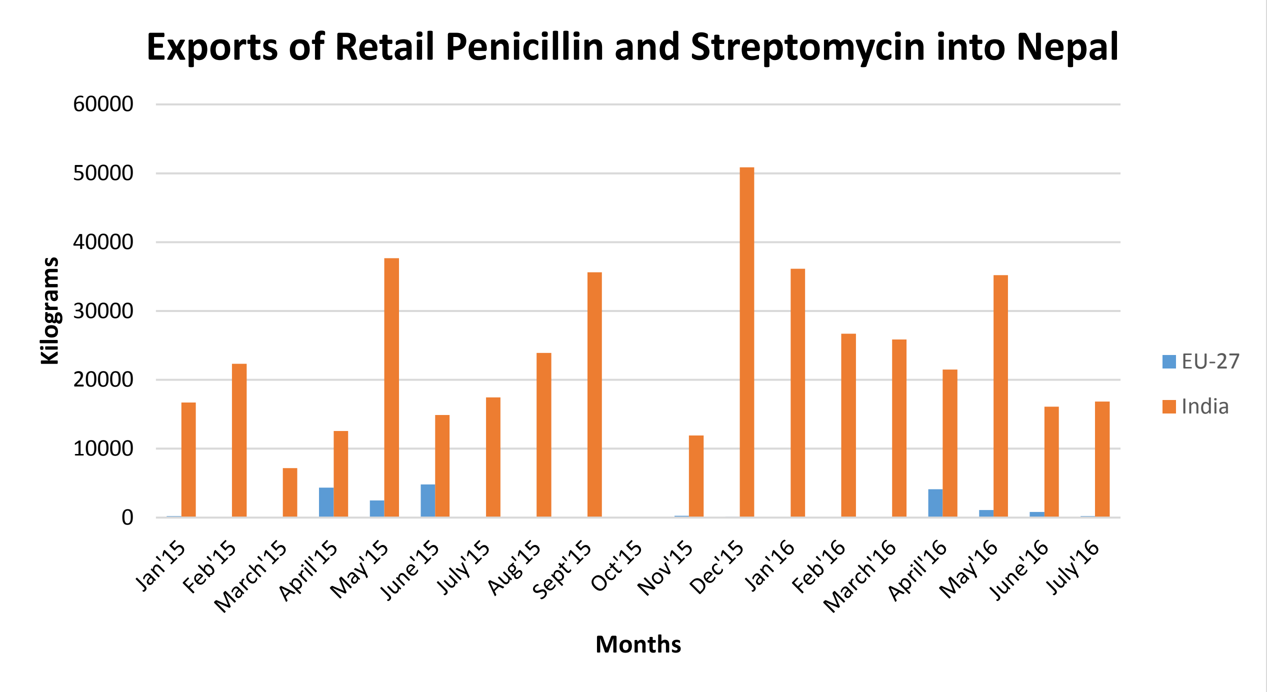

Figure S5. Monthly exports (by weight) of antibiotics Penicillin and streptomycin in dosage form into Nepal before, during and after border blockade

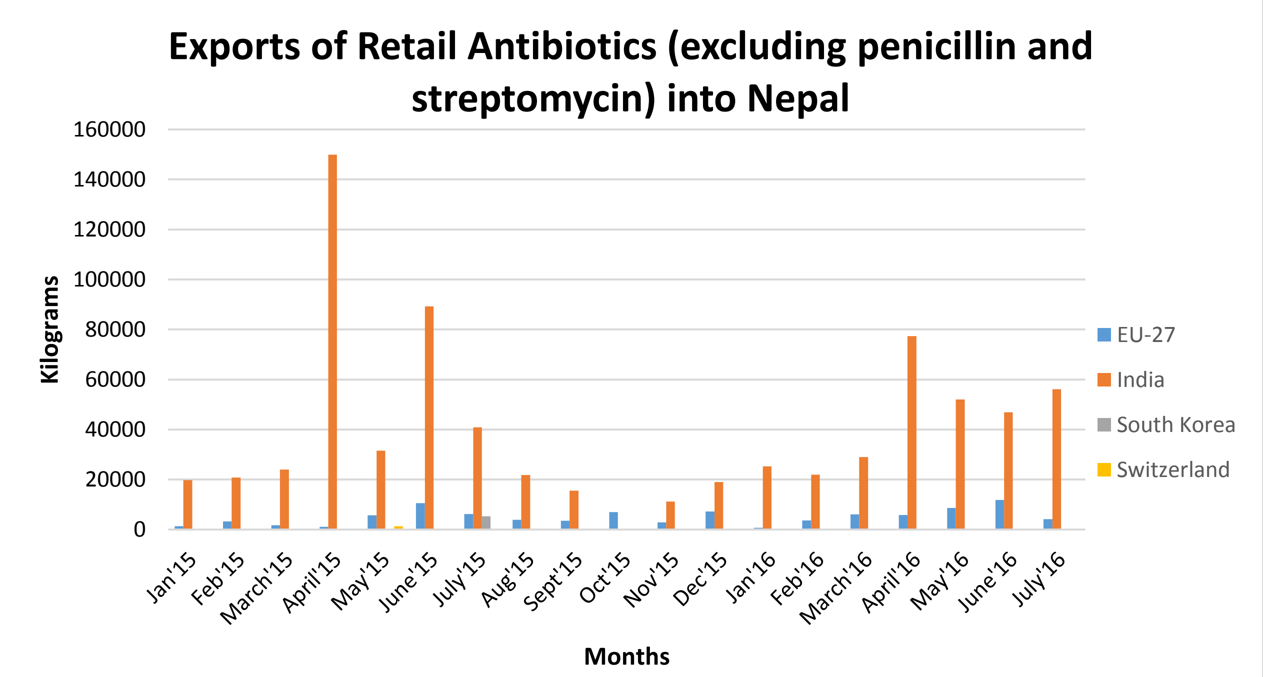

Figure S6. Monthly exports (by weight) of retail antibiotics other than penicillin and Streptomycin into Nepal before, during and after border blockade

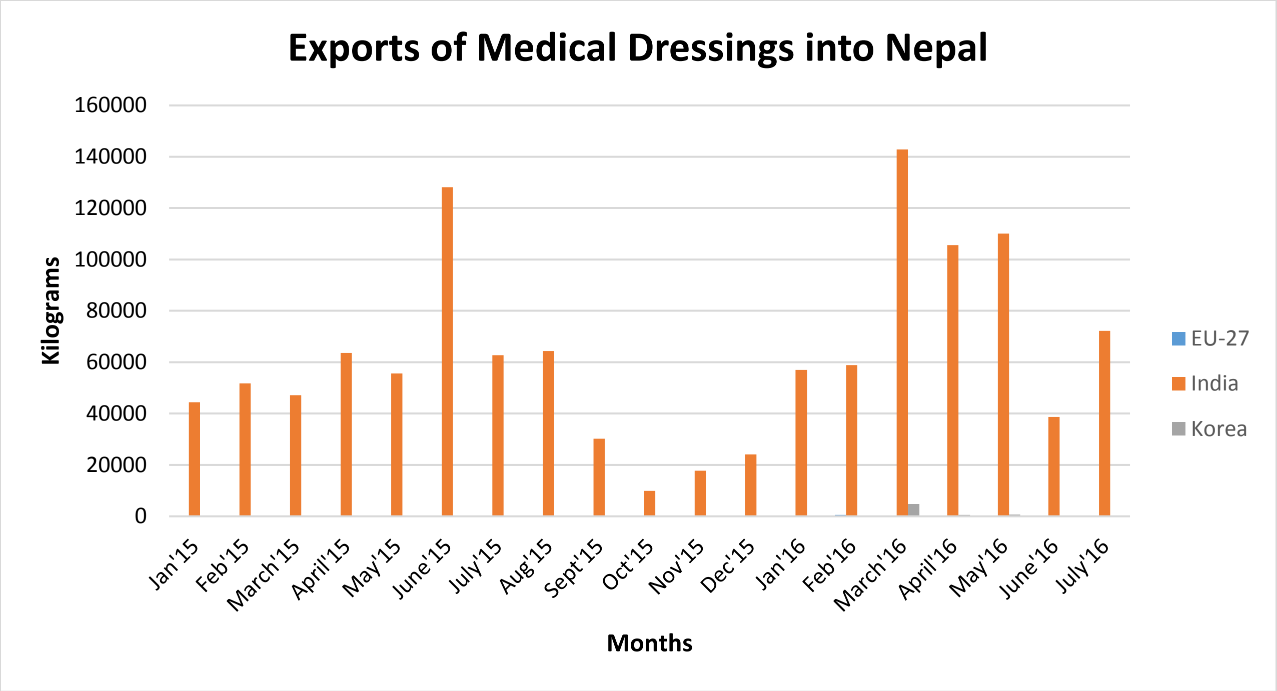

Figure S7. Monthly exports of medical dressings (by weight) into Nepal before, during and after border blockade

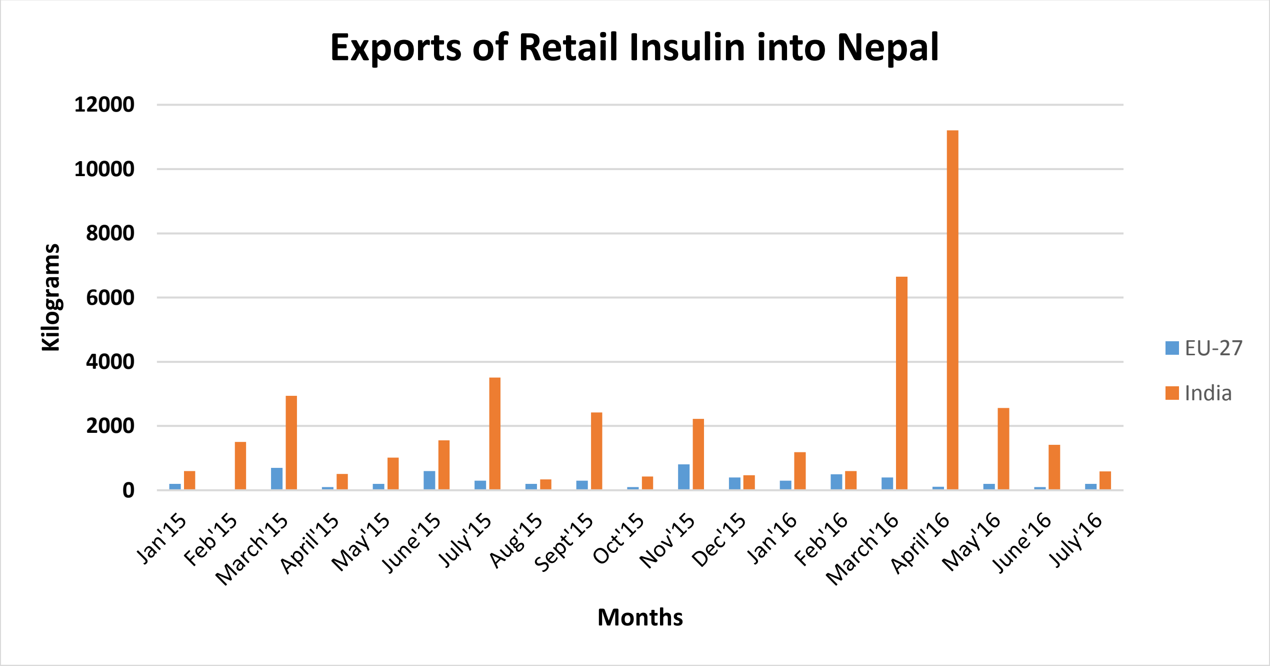

Figure S8. Monthly exports (by weight) of retail insulin into Nepal before, during and after border blockade

Reference:

1. United Nations. International Merchandise Trade Statistics: Compilers Manual, Revision 1 (IMTS 2010-CM). New York, 2013. Available from: http://unstats.un.org/unsd/trade/methodology%20imts.htm (accessed on January 05, 2017)
